# The Osteogenic Potential of Falciform Ligament-Derived Stromal Cells—A Comparative Analysis between Two Osteogenic Induction Programs

**DOI:** 10.3390/bioengineering9120810

**Published:** 2022-12-15

**Authors:** Carla Ferreira-Baptista, André Queirós, Rita Ferreira, Maria Helena Fernandes, Bruno Colaço, Pedro Sousa Gomes

**Affiliations:** 1 Centre for the Research and Technology of Agro-Environmental and Biological Sciences (CITAB), University of Trás-os-Montes and Alto Douro (UTAD), 5000-801 Vila Real, Portugal; 2 BoneLab—Laboratory for Bone Metabolism and Regeneration, Faculty of Dental Medicine, University of Porto, 4200-393 Porto, Portugal; 3 REQUIMTE/LAQV, University of Porto, 4100-007 Porto, Portugal; 4 REQUIMTE/LAQV, Department of Chemistry, University of Aveiro, 3810-193 Aveiro, Portugal; 5 SCIVET-Grupo Breed, 4580-593 Paredes, Portugal; 6 CECAV—Animal and Veterinary Research Centre, University of Trás-os-Montes and Alto Douro (UTAD), 5000-801 Vila Real, Portugal; 7 Associate Laboratory for Animal and Veterinary Sciences (AL4AnimalS), 5000-801 Vila Real, Portugal

**Keywords:** mesenchymal stromal cells, adipose tissue, falciform ligament, osteogenesis, retinoic acid

## Abstract

Mesenchymal stromal cells (MSCs) have gained special relevance in bone tissue regenerative applications. MSCs have been isolated from different depots, with adipose tissue being acknowledged as one of the most convenient sources, given the wide availability, high cellular yield, and obtainability. Recently, the falciform ligament (FL) has been regarded as a potential depot for adipose tissue-derived stromal cells (FL-ADSCs) isolation. Nonetheless, the osteogenic capability of FL-ADSCs has not been previously characterized. Thus, the present study aimed the detailed characterization of FL-ADSCs’ functionality upon osteogenic induction through a classic (dexamethasone-based-DEX) or an innovative strategy with retinoic acid (RA) in a comparative approach with ADSCs from a control visceral region. Cultures were characterized for cell proliferation, metabolic activity, cellular morphology, fluorescent cytoskeletal and mitochondrial organization, and osteogenic activity–gene expression analysis and cytochemical staining. FL-derived populations expressed significantly higher levels of osteogenic genes and cytochemical markers, particularly with DEX induction, as compared to control ADSCs that were more responsive to RA. FL-ADSCs were identified as a potential source for bone regenerative applications, given the heightened osteogenic functionality. Furthermore, data highlighted the importance of the selection of the most adequate osteogenic-inducing program concerning the specificities of the basal cell population.

## 1. Introduction

In recent years, mesenchymal stromal cells (MSCs) have gained special interest for their therapeutic potential in bone regeneration applications [1,2,3]. MSCs are a population of multipotent cells that exhibit a variety of promising characteristics, such as the capacity for self-renewal, fast proliferation, and differentiation into mesenchymal populations [4,5]. The immunosuppressive and immunomodulatory properties of MSCs, as well as the ability to differentiate in response to niche stimulation, make them suitable for use in tissue engineering applications, with particular relevance for bone defect-assisted repair [4,5,6]. MSCs have been successfully used by direct injection or upon biomaterial loading in various clinical applications in human [7,8,9,10] and veterinary applications [11], significantly inducing the bone healing process. In dogs, particularly, notable positive outcomes have been reported from MSCs’ therapies used in bone tissue regeneration and reconstruction [1,12,13].

MSCs in dogs have been isolated from various tissues, such as bone marrow [14,15], adipose tissue [2,3,16], umbilical cord/umbilical cord blood [15,17], and muscle and periosteum [18]. Regarding these, the adipose tissue is acknowledged as one of the most convenient sources of MSCs, given the wide availability, high yield of MSCs upon isolation, and obtainability by minimally invasive procedures [19]. Falciform ligament—a derivative of the embryonic ventral mesentery extending from the liver wall to the diaphragm—has been recently regarded as a potential depot for adipose tissue-derived stromal cells (ADSCs) isolation [20,21,22,23]. It can be simply obtained during routine interventions, such as castration and ovariohysterectomy in dogs, through a small incision [24], requiring less surgical dissection than the harvest of adipose tissue from other anatomical locations [25]. Furthermore, falciform ligament-derived ADSCs (FL-ADSCs) have been shown to exhibit a higher proliferation capacity and a greater number of surface markers [24,26,27]. However, little is known regarding the differentiation capacity of this population, and to the best of the authors’ knowledge, no studies have characterized the osteogenic differentiation capability of FL-ADSCs.

Accordingly, in this research, we investigated the functional activity and osteogenic functionality of FL-ADSCs, in a comparative approach with ADSCs isolated and grown from a representative visceral region, for prospective applications in bone regenerative medicine applications. The osteogenic differentiation capacity of ADSCs has been extensively studied since 2001 [28]. Since then, most applications, either experimental or within the frame of therapeutic applications, have relied on osteogenic induction through the supplementation of the culture medium with dexamethasone, β-glycerophosphate, and ascorbic acid [2,13,29]. Most recently, several studies with rat-, mouse-, and cat-derived cells have reported the ability of retinoic acid (RA) to promote osteogenic differentiation [30,31,32]. In dog-derived cells, so far, only one study evaluated the RA-mediated osteogenic induction of ADSCs, focusing exclusively on cells from the subcutaneous depot [33], with no available data on visceral-derived populations. Accordingly, this research aimed to characterize the functionality of ADSCs isolated and grown from the falciform ligament, induced into the osteogenic program through either dexamethasone, β-glycerophosphate, and ascorbic acid (DEX), or retinoic acid (RA)—in a comparative approach with the functionality of ADSCs isolated and grown from a representative visceral region.

## 2. Materials and Methods

### 2.1. Animals

Samples of adipose tissue were obtained from 6 healthy dogs (6 months–3 years, within the ideal interval of body weight [34]) during routine castration or ovariohysterectomy. The adipose tissue was obtained from the falciform ligament and periovarian visceral region. The experimental protocol was approved by the University of Trás-os-Montes e Alto Douro (UTAD) ethical committee—reference 15-CE-UTAD-2021. All animal owners signed the informed consent for the collection of adipose tissue.

### 2.2. Adipose Tissue Histological Characterization

Samples were fixed for 48 h in 10% buffered formaldehyde, then embedded in paraffin and sectioned serially and longitudinally. Five sections of each tissue, 3 μm thick, were stained with hematoxylin and eosin (H&E) for the visualization on a digital virtual microscope (Olympus BX-51/22), with images created using OlyVIA 2.1 software (Olympus Soft Imaging Solutions, GmBH, Münster, Germany).

### 2.3. Isolation of ADSCs

Adipose tissue samples were decontaminated, as previously described [30]. Subsequently, samples were cut into 1–3 mm^3^ fragments and digested for 40 min, at 37 °C, with 0.1% collagenase type IA (Sigma-Aldrich) in phosphatase-buffered saline (PBS; Sigma-Aldrich). The digested tissue was filtered and centrifuged at 229 *g* for 10 min. After supernatant removal, the obtained cell pellet was resuspended in a culture medium (basal medium, BM) composed of α-MEM, fetal bovine serum (FBS; 10%), streptomycin (100 µg/mL), penicillin (100 UI/mL), and amphotericin B (100 µg/mL), and then seeded at 5 × 10^3^ cells/cm^2^ in cell culture flasks (Corning). Cultures of ADSCs were grown until a confluence of around 80% (≈7 days) was reached. Cells were detached from the culture surface with TrypLE (Gibco) and seeded at 10^3^ cells/cm^2^ for subsequent characterization. Cells of the first subculture were characterized for the expression of surface markers CD29, CD44, CD45, and CD90. The expression rate for CD29, CD44, and CD90 was found to be >95% and <5% regarding CD45. The following antibodies were used: anti-CD29-PE (303004), anti-CD44-FITC (338803), anti-CD45-PE (304007), anti-CD90-FITC (328107), and their respective isotype controls, all from BioLegend. Quantification was conducted in a BD LSRFortessa system. All subsequent assays were conducted on the first subculture cells.

### 2.4. Osteogenic Differentiation of ADSCs

ADSCs’ cultures were grown in two osteogenic media: (i) DEX: α-MEM, penicillin (100 UI/mL), amphotericin B (100 µg/mL), streptomycin (100 µg/mL), FBS (10%), dexamethasone (10 nM; Sigma-Aldrich), ascorbic acid (50 µg/mL; Sigma-Aldrich), and β-glycerophosphate (10 mM; Sigma-Aldrich); or (ii) RA: α-MEM, FBS (10%), streptomycin (100 µg/mL), penicillin (100 UI/mL), amphotericin B (100 µg/mL), and retinoic acid (2.5 µM). Basal medium—BM was used as a control. The medium was changed twice a week during the cell culture experiments.

### 2.5. Cell Proliferation

After cell lysis with Triton X-100 (0.1%; Sigma-Aldrich), DNA content was quantified according to the manufacturer’s instructions using the Quant-iT™ PicoGreen™ dsDNA assay Kit (Invitrogen, Thermo Fisher Scientific, Waltham, MA, USA). Fluorescence was measured at selected wavelengths: excitation (480 nm) and emission (528 nm), in a microplate reader (Synergy HT, BioTek).

### 2.6. Metabolic Activity

Cultures’ metabolic activity was established by the MTT (3-(4,5-dimethylthiazol-2-yl)-2,5-diphenyltetrazolium bromide) assay on days 3 and 6. MTT solution (5 mg/mL) was added, and the samples were incubated for 3 h at 37 °C in a humidified atmosphere with 5% CO_2_. The reduction in MTT led to the formation of purple formazan products by viable cells. Then, formazan crystals were dissolved, and the absorbance was determined at 550 nm in a microplate reader (Synergy HT, BioTek).

### 2.7. Immunostaining of F-Actin Cytoskeleton, Mitochondria, and Nucleus

The cytoskeletal organization and mitochondrial morphology were evaluated after 4 days of culture. To achieve this purpose, live cells were labeled with MitoSpy™ Red (250 nM, Biolegend, San Diego, CA, USA). Then, cells were fixed for 10 min in 3.7% formaldehyde (Sigma-Aldrich) and washed twice with PBS. Cultures were stained, for 30 min, with Alexa Fluor-conjugated phalloidin (Alexa Fluor^®^ 488 Phalloidin: Molecular Probes) in 1% BSA/PBS for F-actin imaging and for 15 min with Hoechst 33,342 (8 μg/mL; Sigma-Aldrich) for nucleus counterstaining. Cells were imaged in a digital imaging system (Celena S, Logos Biosystems). For the determination of cell area and cell skewness (i.e., the ratio of the cell width over cell length), 20 representative fields were digitally quantified using the ImageJ software (version 1.51).

### 2.8. Gene Expression Assessment

Cell cultures were characterized by real-time quantitative polymerase chain reaction (RT-qPCR) to assess osteogenic-related gene expression on day 7. Total RNA was extracted according to the manufacturer’s instructions, using TRIzol^TM^ reagent (Invitrogen). The purity and concentration of the total RNA in each sample were assessed by UV spectrophotometry by the determination of the A260 nm/A280 nm ratio. RNA was reverse transcribed and amplified with the NZY First-Strand cDNA Synthesis Kit (Nzytech), according to the manufacturer’s instructions. Used primers, presented in Table 1, were provided and validated by Bio-Rad Laboratories (Bio-Rad Laboratories, Inc.). The expression of the genes was quantitatively determined on a thermocycler (CFX96^TM^, Bio-Rad) using iQ^TM^ SYBR^®^ Green supermix (Bio-Rad), and data were presented normalized to the control.

### 2.9. Cytochemical Staining of Collagen

The cultures were fixed for 10 min with 1.5% glutaraldehyde in 0.14 M sodium cacodylate buffer. Subsequently, samples were covered for 1 h with Sirius Red dye (0.01 g of Sirius Red F3B per 100 mL of picric acid) and rinsed with 0.01 N HCl to remove all the excess dye. Red-stained collagen deposits were visualized in an inverted phase contrast microscope (Nikon TMS). Representative fields (10) were identified and registered for collagen intensity determination using the ImageJ software (version 1.51).

### 2.10. Cytochemical Staining of Alkaline Phosphatase

The cultures were fixed (1.5% glutaraldehyde in 0.14 M sodium cacodylate buffer, 10 min) and covered, for 1 h, with Tris buffer (pH 10) containing sodium naphthyl phosphate and Fast Blue RR salt (both from Sigma-Aldrich). They were subsequently rinsed with distilled water and air-dried. Stained cultures, with a staining variation from brown to black according to the enzyme activity, were photographed in an inverted phase contrast microscope (Nikon TMS). Ten representative fields were registered for alkaline phosphatase intensity determination using the ImageJ software (version 1.51).

### 2.11. Statistical Analysis

Obtained data were analyzed by the JMP^®^ Statistics software (7v), and the normality (Shapiro–Wilk) and heterogeneity of treatment variances were performed before analyzing the data. Group comparison was performed using two-way ANOVA, followed by Tukey’s post hoc. Values of *p* < 0.05 were considered significant. Data were presented as mean ± standard deviation.

## 3. Results

### 3.1. Histological Analysis of Adipose Tissue

**Figure 1:**
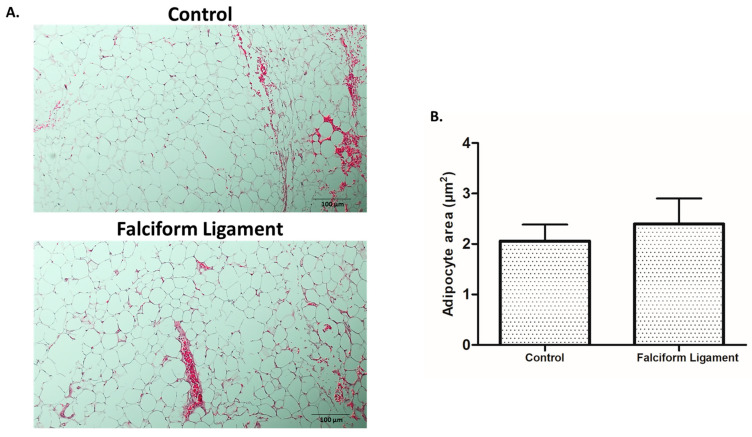
Histological analysis of adipose tissue samples harvested from the control and falciform ligament depots. (**A**). Illustrative images of adipose tissue stained with hematoxylin–eosin (scale bar: 100 µm; magnification 10×). (**B**). Histogram of the mean adipocyte area in the deposits of the control and the falciform ligament (n = 30).

The adipose tissue samples from the falciform ligament and the representative visceral region (control) were characterized histologically upon hematoxylin/eosin staining (Figure 1A). Adipocytes in both tissues were arranged in close contact with each other in a high cell density distribution, with tissues exhibiting a thorough interspersed vascular network, with vessels of distinct caliber—either microvessels or larger caliber structures—as evidenced in Figure 1A. Quantitative analysis of the adipocytes’ area revealed no significant differences between both tissues (Figure 1B).

### 3.2. Cell Proliferation

**Figure 2:**
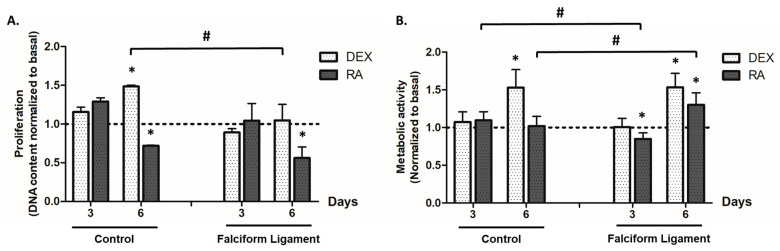
Cell proliferation (**A**) and metabolic activity (**B**) of ADSCs grown from the control and falciform ligament depots. * Significantly different from basal (*p* < 0.05). # Significantly different between the control and the falciform ligament (*p* < 0.05). Dexamethasone, β-glycerophosphate, and ascorbic acid-supplemented media (DEX), retinoic acid-supplemented media (RA).

ADSCs from the falciform ligament and control tissue, grown in the presence of distinct osteogenic-inducing conditions, presented a dissimilar proliferation activity as compared to basal conditions—the absence of osteogenic inducers (Figure 2A). DEX significantly increased cell proliferation in ADSCs from the control tissue on day 6. In the presence of RA, a trend for reduced proliferation was attained, with significantly lower levels being attained on day 6 for cultures from both tissues.

### 3.3. Metabolic Activity

The metabolic activity was estimated by the MTT assay, as shown in Figure 2B. DEX induction was found to increase the cultures’ metabolic activity at day 6, for both the control- and falciform ligament-derived populations. RA induction only increased MTT reduction values in FL-ADSCs, on day 6, as compared to basal.

### 3.4. Immunostaining of F-Actin Cytoskeleton, Mitochondrial, and Nucleus

**Figure 3:**
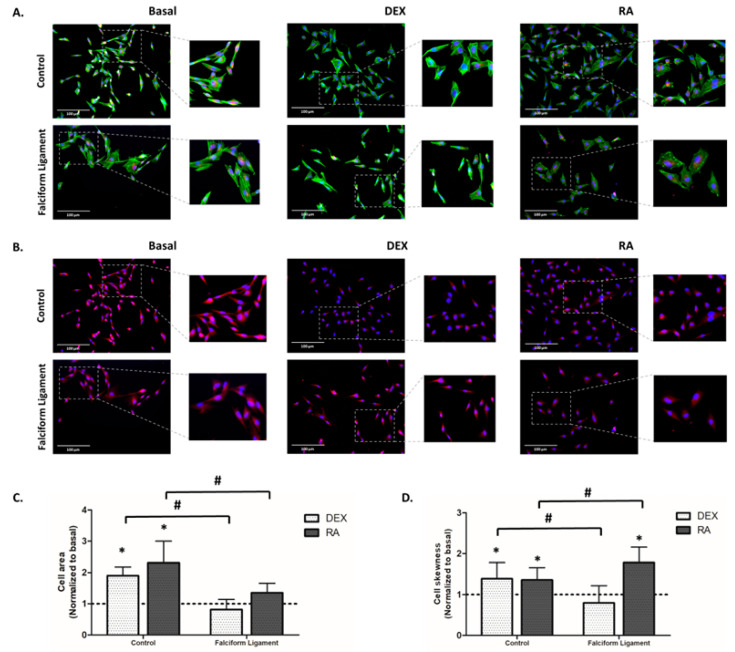
Representative images of ADSCs grown from the control and the falciform ligament (4 days) regarding cellular morphology (**A**) and mitochondrial morphology and distribution (**B**). Mitochondria = red; filaments of F-actin = green; and nucleus = blue. The scale bar corresponds to 100 μm. Cell area (**C**) and cell skewness (**D**). * Significantly different from basal (*p* < 0.05). # Significantly different between the control and the falciform ligament (*p* < 0.05).

The cytoskeletal organization and mitochondrial morphology of ADSCs’ cultures were characterized by fluorescence staining of the F-actin cytoskeleton and mitochondrial tracking, respectively. From analyzing the immunostained images in the basal condition, it was possible to observe that cells grown from the falciform ligament and the control exhibited similar morphological characteristics - an elongated fibroblast-like morphology, well-marked stress fibers, and a defined F-actin cytoskeleton (Figure 3A), as well as a distributed and continuous mitochondrial network with a perinuclear distribution (Figure 3B). However, it was found that cells grown from the falciform ligament exhibited a more elongated morphology than those grown from the control in basal conditions (Figure 3A). Comparatively, cultures grown in the presence of DEX showed a more intense F-actin stain than cells grown in the presence of RA or basal conditions (Figure 3A). However, cultures in the presence of RA exhibited a more elongated morphology and a larger cytoplasmic volume than cells grown in the presence of DEX and basal (Figure 3A). The results of the cell area (Figure 3C) and cell skewness (Figure 3D) confirmed these observations, with cells exposed to RA showing significantly higher values than those grown in DEX and basal.

### 3.5. Gene Expression Analysis

**Figure 4:**
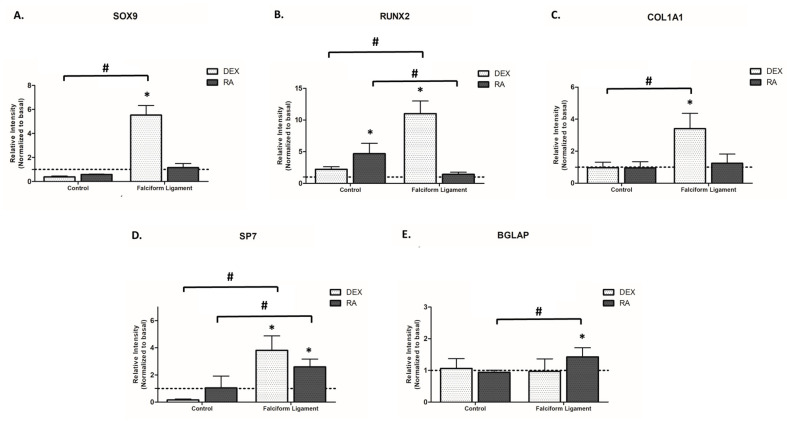
Analysis of the gene expression, by RT-qPCR, of osteogenic differentiation markers such as SOX9 (**A**), RUNX2 (**B**), COL1A1 (**C**), SP7 (**D**) and BGLAP (**E**), in ADSCs of the control and falciform ligament after 7 days of subculture. * Significantly different from basal (*p* < 0.05). # Significantly different between the control and the falciform ligament (*p* < 0.05).

ADSCs cultures established from the falciform ligament and control showed a dissimilar gene expression profile when grown in distinct osteogenic conditions (Figure 4). Analyzing the results of SOX9, the master regulator to determine undifferentiated mesenchymal progenitors’ commitment to osteochondroprogenitors, an increased expression was attained in FL-derived populations with DEX induction, while RA induced no significant differences in cultures from both origins (Figure 4A). Regarding RUNX2, an earlier master regulator of the osteogenic commitment, it is noticeable that DEX significantly induced the expression in cultures grown from the falciform ligament, while RA significantly increased the expression in cultures from the control tissue, as compared to basal conditions (Figure 4B). Regarding the assessment of COL1A1, an early marker of osteoprogenitor cells coding for the major protein component of the bone extracellular matrix [35,36], DEX significantly induced the expression in FL-derived populations, while no significant differences were observed with RA induction (Figure 4C). DEX and RA further induced SP7 expression (a late osteogenic transcription factor) in falciform ligament-derived cultures, while no significant differences were attained in control-derived cultures (Figure 4D). In addition, lastly, regarding BGALP, a late marker of developing osteoblasts coding for osteocalcin that regulates bone remodeling and energy metabolism [35,36], RA was found to induce its expression in falciform ligament cultures, as compared to basal, with no significant differences in control-derived cultures for both osteogenic-inducing programs (Figure 4E). Overall, there was an established trend for FL-derived cultures to express significantly higher levels of the assayed genes, particularly upon DEX exposure.

### 3.6. Cytochemical Staining of Collagen

**Figure 5:**
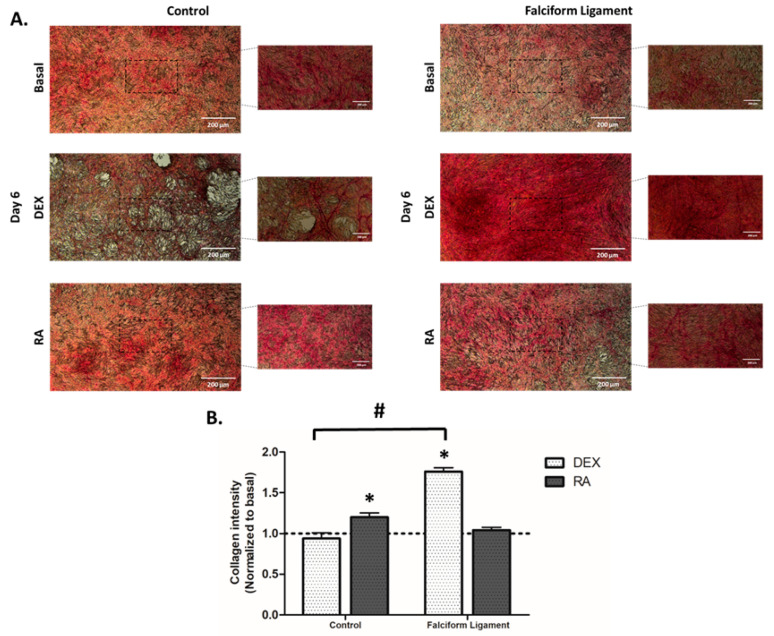
(**A**). Illustrative images of ADSCs cultures established from the control and falciform ligament, stained for the presence of collagen. Scale bar: 100 μm; magnification 2.5× and 5×. (**B**). Histogram of the mean staining intensity on the control- and falciform ligament-derived cultures (n = 10). * Significantly different from basal (*p* < 0.05). # Significantly different between the control and the falciform ligament (*p* < 0.05).

The expression of collagen within the culture system was assessed after staining with Sirius Red (Figure 5A). From analyzing the images representative of 6-day cultures, it is possible to verify differences in the staining pattern, being noteworthy the high cell density and the nodular arrangement, particularly in osteogenic-induced conditions. In control-derived cultures, significantly higher staining was attained with RA, while for FL-derived populations, an increased culture staining was attained upon DEX induction. Quantitative analysis of the culture staining intensity (Figure 5B) supports the qualitative findings.

### 3.7. Cytochemical Staining of Alkaline Phosphatase

**Figure 6:**
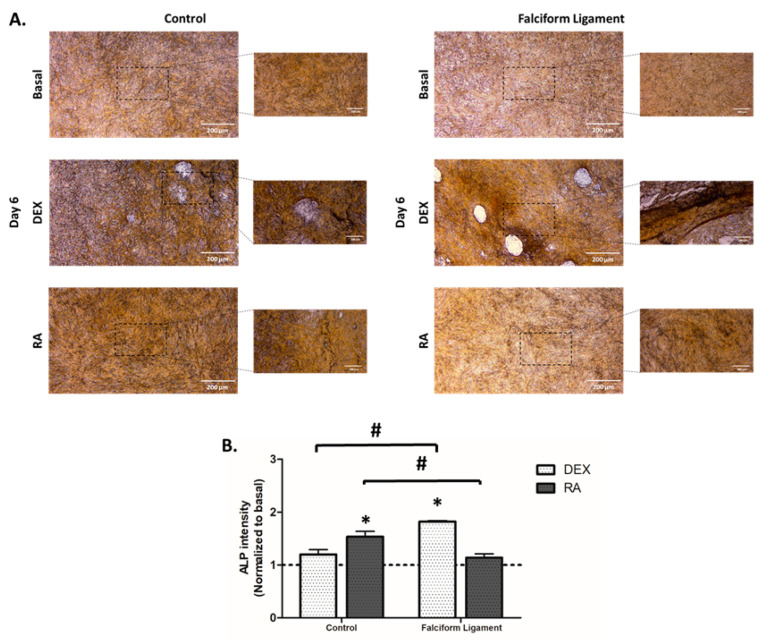
(**A**). Illustrative images of ADSCs cultures established from the control and falciform ligament, stained for the activity of ALP. Scale 100 μm. Magnification 2.5× and 5×. (**B**). Histogram of the mean staining intensity of the control- and falciform ligament-derived cultures (n = 10). * Significantly different from basal (*p* < 0.05). # Significantly different between the control and the falciform ligament (*p* < 0.05).

ALP was evaluated by a cytochemical technique (Figure 6A). Cultures grown for 6 days presented a high cell density and a high expression of ALP, especially evident in areas of high cell density with the formation of nodular structures, characteristic of the osteoblastic phenotype. Comparatively, in control-derived ADSCs’ cultures, RA induced the highest enzyme activity, while for FL-derived cultures, DEX was found to induce the highest activity. The quantitative assessment of the intensity of enzyme activity (Figure 6B) substantiates the described findings.

## 4. Discussion

Bone lesions are one of the most common orthopedic conditions in veterinary medicine, particularly in dogs [37,38,39], being associated with various etiologies such as falls, traffic accidents, crushing injuries, animal biting, and human aggression [37,38,39,40,41,42,43]. Although there are currently several therapeutic approaches for successful bone healing, these have been associated with established complications such as failure of fixing and pin migration, infection, and disturbance/impairment of the healing process, converging to increased morbidity, animal suffering, and increased treatment cost [38,44]. In this regard, the development of innovative therapies that enhance bone healing, and promote and stimulate the regenerative process, is of the utmost relevance, particularly when a translational link to human medicine can be established.

ADSCs have been investigated as an important cell source for bone regenerative therapies, with a reported efficacy in fracture healing and injury reduction, demonstrating enhanced outcomes in bone regeneration [45]. In this regard, the falciform ligament has recently been considered a potential depot for the isolation of ADSCs since it can be easily obtained during routine surgical procedures [24]. However, to the best of our knowledge, no previous studies have evaluated the osteogenic capacity of FL-ADSCs. In this sense, it is of the most importance to analyze the osteogenic functionality of FL-ADSCs in a comparative approach with cultures established from a representative visceral region to address the potential interest of this population in bone-related regenerative applications.

In the various studies conducted in dogs’ cells so far, osteogenic induction of ADSCs has focused on the use of a culture medium, DEX, used in this study [2,16,29,46,47,48,49]. Recently, Levi et al. demonstrated in a study with dog-derived populations the innovative application of RA in osteogenic induction, focusing, however, exclusively on cells isolated from the subcutaneous adipose depot [33], with no available data on populations in the visceral region. In this line, this study further aims to characterize the osteogenic capability of FL-ADSCs, compared to that of ADSCs grown from a representative visceral region, induced either by the classic osteogenic induction (DEX) or with RA.

Regarding the morphological evaluation of the adipose tissue isolated from the falciform ligament and the control visceral location, no significant differences were found between the two harvested tissues, namely regarding the mean adipocyte area and vascularization. This is consistent with previous data from mice and humans, which showed that tissues collected from the same region have identical characteristics [50,51,52,53]. Despite the absence of morphological differences, previous reports highlight a distinctive cell functionality, with non-induced FL-ADSCs expressing a wide range of surface markers and an increased proliferation capacity than ADSCs from other anatomical locations [25,27]. Accordingly, and in the absence of the functional evaluation of FL-ADSCs in osteogenic-inducing conditions, a thorough assessment was established.

The evaluation of cell proliferation and metabolic activity showed a similar pattern between cultures established from falciform ligament and control tissues, despite the differences induced by both osteogenic inducers. Briefly, there was a trend for increased proliferation and metabolic activity in DEX-induced cultures over time, while with the RA exposure, a trend for reduced proliferation through time was attained. This enhanced response to DEX exposure is thought to be associated with the activation of early response transcription factors, such as activator protein 1 (AP-1), which induces proliferation by positively regulating the expression of genes such as D-type cyclins [54]—known to modulate cyclin-dependent kinases (Cdk) with a determinant activity in the passage from the G1 to the S phase of the cell cycle [55,56]. Previous reports substantiate the present findings, with DEX-induction actively inducing the proliferation of ADSCs [57,58]. Contrariwise, in the presence of RA, a reduction in the proliferation is expected, which is thought to be related to a decreased expression in Cdk2, Cdk4/Cdk6, D-type cyclins (D1, D2, and D3), and cyclin E, which suppress the progression from the G1 to the S phase, thus hindering cell proliferation [56,59]. A similar trend has been reported in studies conducted on ADSCs from distinct species exposed to RA, namely those grown from rats, cows, and cats [30,31,57].

ADSCs’ cultures, regardless of the tissue of origin, exhibited a fibroblastic-like arrangement with an elongated morphology, and well-marked F-actin organized into evident stress fibers when grown in basal conditions. This morphology is characteristic of ADSCs obtained from dogs [2,3,58,60] and other species, namely rats, mice, cats, horses, rabbits, pigs, and humans [3,30,61,62,63,64,65]. In a comparative approach relative to cultures grown in the presence of the osteogenic inducers, more elongated cell morphology and an increased cytoplasmic area were verified in cells cultured in RA, compared to those cultured in DEX. A similar trend was observed previously in studies with cat- and mouse-derived cells [30,66]. Despite the fact that the mechanism by which cells cultured in RA exhibit the reported differences is not fully described, it is thought to be related to alterations in the cytoskeletal arrangement, attributed to an increased thickness of actin stress fibers promoted by the MAPK signaling activation [67,68].

To evaluate the osteogenic differentiation process of the established cultures, the gene expression of early (i.e., SOX9, RUNX2, and COL1A1) and late (i.e., SP7 and BGLAP) markers was assayed and seconded by the cytochemical assessment of collagen and alkaline phosphatase. SOX9 is the master transcription factor to determine undifferentiated mesenchymal progenitors’ commitment to osteochondroprogenitors. RUNX2 is the major osteogenesis-related transcriptional regulator, further inducing the expression of distinct downstream effectors as collagen type I—the major structural protein of the bone tissue. ALP, an enzyme that cleaves pyrophosphate to produce inorganic phosphate and free calcium, plays a major role in the matrix mineralization process [69,70]. Briefly, our data showed a significantly higher expression of SOX9 (≈6 fold), RUNX2 (≈10 fold), and COL1A1 (≈4 fold) in cultures established from the falciform ligament, grown in the presence of DEX, as compared to those grown in basal conditions, additionally outperforming RA induction. A similar tendency was verified for the expression of the late osteogenic transcriptional regulator SP7 [69,71,72], with the highest levels being attained in the presence of DEX. Consistently, cytochemical analysis of collagen and ALP expression revealed the reported trend, increased staining and the formation of nodular arrangements in cultures grown from the falciform ligament in DEX, evidencing the increased osteogenic commitment [69,70].

Whether no previous studies have evaluated the osteogenic induction of falciform-derived populations, DEX-mediated osteogenic commitment has been regarded as an effective approach in dog-derived ADSCs when used at low concentrations (<100 nM), as presently verified [2,16,29]. Mechanistically, DEX promotes the osteogenic program via a RUNX2 upregulation through distinct signaling, as through the WNT/β-catenin and MAPK pathways, in a process associated with FHL2 induction [73]. Additionally, and contrariwise to the present findings, DEX-induction has been associated with the downregulation of SOX9—a required step for the upregulation of RUNX2 and prosecution of the osteogenic commitment [74], at least in bone marrow-derived stromal cells (BMDSCs) [75]. Verified differences in cell responsiveness regarding SOX9 may be related to the dissimilar osteogenic gene expression profile of the undifferentiated populations, as ADSCs, compared to BMDSCs, express significantly higher baseline levels of SOX9 [76]. Accordingly, the inhibition of SOX9 in ADSCs downregulated the expression of osteogenesis-related genes (i.e., DLX3, p300, RUNX2, COL1A1, and VEGFα), substantiating the inductive capability of SOX9 to enhance the osteogenic commitment of ADSCs, as presently verified for FL-derived cells upon DEX induction. Furthermore, an increased SP7 expression and cytochemical evidence of increased collagen and ALP activity were attained, in line with the increased osteogenic commitment [77,78].

In cultures grown from the control, a significantly higher expression of RUNX2 and, as well, increased collagen and ALP staining were attained in RA-induced cultures, as compared to those induced with DEX. RA is known to have pleiotropic effects over osteogenic populations, with distinctive modulatory capabilities in cells of distinct origins [79,80]. Previous reports show that RA displays a distinctive osteogenic-inducing capability in subcutaneous ADSCs, significantly enhancing the commitment of rat cell populations, displaying limited effectiveness on canine cells, and negatively influencing human populations [33]. Our research group has further demonstrated the RA efficacy in inducing the osteogenic commitment of cat-derived ADSCs [30]. RA induction of osteogenesis within ADSCs seems to occur via the bone morphogenetic protein (BMP) signaling pathway through the increased signaling and upregulation of BMP receptors [32,81]. The increased signaling seems to converge into the enhanced phosphorylation of SMAD proteins, able to upregulate target genes that control the osteogenic commitment [30,82]. In the present study, RA was found to significantly increase the expression of distinct osteogenic markers—including a major transcription factor of the osteogenic program, and the cytochemical staining of specific osteogenic markers (i.e., ALP and collagen), as compared to basal conditions, supporting the reported efficacy on ADSCs [69,70]. Nonetheless, differences between cell populations were attained, with DEX induction outperforming the osteogenic induction of RA in FL-ADSCs. Differences in the cellular functionality may relate to eventual distinctive gene expression profiles of visceral-derived populations, as previously reported for ADSCs grown from subcutaneous and visceral regions [83,84,85] and, as well, regarding the functional activity of the tissue depots used for cell isolation [52,86,87].

## 5. Conclusions

This study addressed, for the first time, and to the best of the authors’ knowledge, the osteogenic potential of ADSCs isolated and grown from the falciform ligament in a comparative way to a control visceral adipose tissue depot. FL-ADSCs presented an increased osteogenic potential as compared to ADSCs from the visceral control depot, particularly when induced with DEX. RA significantly induced the osteogenic capability of the cells from both origins but was particularly effective in the enhancement of those from the control. Overall, FL-ADSCs may be regarded as a relevant cell source for bone-related applications, given the high osteogenic capability and ease of access during routine surgical procedures. Present data further highlights the importance of the selection of the most adequate osteogenic-inducing program concerning the specificities of the cell population.

## Figures and Tables

**Table 1:** Genes and assay ID registers used for the gene expression analysis.

Gene	Assay ID
Beta-actin (*ACTB*)	qCfaCED0037901
SRY-Box transcription factor 9 (*SOX9*)	qCfaCED0025675
RUNX family transcription factor 2 (*RUNX2*)	qCfaCED0033695
Collagen type I alpha 1 chain (*COL1A1*)	qCfaCED0027854
Osterix (*SP7*)	qCfaCED0032017
Osteocalcin (*BGLAP*)	qCfaCED0031563

## Data Availability

The datasets generated during and/or analyzed during the current study are available from the corresponding author on reasonable request.
